# Damage Evolution and Fracture Behavior of C/SiC Minicomposites with Different Interphases under Uniaxial Tensile Load

**DOI:** 10.3390/ma14061525

**Published:** 2021-03-20

**Authors:** Zhongwei Zhang, Longbiao Li, Zhaoke Chen

**Affiliations:** 1 Institute of Advanced Structure Technology, Beijing Institute of Technology, Beijing 100081, China; 2 College of Civil Aviation, Nanjing University of Aeronautics and Astronautics, No. 29 Jiangjun Ave., Nanjing 211106, China; 3 State Key Laboratory of Powder Metallurgy, Central South University, Changsha 410083, China; chenzhaoke2008@csu.edu.cn

**Keywords:** ceramic matrix composites (CMCs), C/SiC, minicomposite, matrix cracking, interface debonding, fiber pullout

## Abstract

In this paper, the tensile damage and fracture behavior of carbon fiber reinforced silicon carbide (C/SiC) minicomposites with single- and multiple-layer interphases are investigated. The effect of the interphase on the tensile damage and fracture behavior of C/SiC minicomposites is analyzed. The evolution of matrix cracking under the tensile load of the C/SiC minicomposite with a notch is observed using the digital image correlation (DIC) method. The damage evolution process of the C/SiC minicomposite can be divided into four main stages, namely, (1) an elastic response coupled with partial re-opening of thermal microcracking; (2) multiple matrix microcracking perpendicular to the applied loading; (3) crack opening and related fiber/matrix, bundle/matrix, and inter-bundle debonding; and (4) progressive transfer of the load to the fibers and gradual fiber failure until composite failure/fracture. On the fracture surface, a large number of fibers pulling out of the samples with both single-layer and multi-layer interphases can be clearly observed.

## 1. Introduction

In the aerospace field, C/SiC ceramic matrix composites (CMCs) are important thermal protection and thermal structure materials, which have been widely used in the nose cone and wing leading edge of near-space vehicles, rocket engine nozzles, advanced aircraft control rudders, and other components [1,2,3]. With the demand for hypersonic vehicles for the integrated structure of load bearing and reliability, the production of higher mechanical properties (i.e., strength and toughness) of C/SiC composites is an urgent matter. During the fracture process of C/SiC composites, the internal structure consumes most of the fracture energy by inducing microcrack deflection, fiber fracture, and pulling out from the matrix, which improves the flexural strength and fracture toughness of the monolithic ceramic. The interphase is an important phase for the design of the C/SiC composite system and the guarantee of high mechanical properties [4,5,6,7,8]. If the interface bonding force between the reinforcement and matrix is weak, it is difficult to realize the stress transfer, and for strong interface boning, the improvement to the composite’s strength and toughness is not obvious [9,10]. Jimenez et al. [11] fabricated siloxane precursor-based protective coatings for high-modulus carbon fibers in CMCs. It was found that the enhanced fiber/matrix interface strength further improved the mechanical performance of the fabricated composites. Jimenez et al. [12] investigated the effect of fiber treatments on the interfacial bonding strength of the carbon fiber reinforced AlSi5 composites. Composites from coated and untreated fibers are characterized via the fiber push-out technique. Glassy carbon and silicon oxycarbide coatings lead to a reduction of the interfacial bonding strength of 81% and 83%, respectively. A fractographic study of the tested specimens by means of scanning electron microscopy (SEM) and energy dispersive X-ray analysis (EDX) identified the adhesive failure at the fiber/coating contact as the predominant failure mode of the composite. To realize the scientific design of the interphase and the high mechanical properties of materials, researchers need to identify the “hidden” factors that restrict the performance of the interphase first; quantitatively characterize and evaluate these parameters; reveal the internal mechanism of the interphase function from a deeper perspective; and establish the methods of the interphase performance test, characterization, and theoretical prediction.

For the C/SiC composite, the fracture strain of the SiC matrix (i.e., *ε*_m_ < 0.1%) is far less than that of carbon fiber (i.e., *ε*_f_ ≈ 1%), and the modulus of the SiC matrix (i.e., *E*_m_ ≈ 350 GPa) is far higher than that of carbon fiber (i.e., *E*_f_ = 230–294 GPa) [12,13]. Under tensile loading of the C/SiC composite, if no interphase debonding or sliding occurs, and the composite strain equals the strain of the carbon fiber and SiC matrix, the SiC matrix fractures at low applied stress, which leads to brittle fracture of the composite. However, if incorporating the interphase between the fiber and the matrix and optimizing the design of the carbon fiber, SiC matrix, and interphase, the C/SiC composite can overcome brittle fracture and exhibit obvious toughness behavior. The interphase of composite materials refers to the zone with significant changes in chemical composition between the matrix and the reinforced phase [14,15]. Many researchers performed investigations on controlling the performance of the interphase by adjusting the composition, microstructure, and thickness. Chen et al. [16] fabricated a multi-layer SiC/TaC gradient distribution interphase into the C/C composite using the chemical vapor infiltration (CVI) method and obtained the hardness and modulus of the interphase through nano-indentation testing. Carrere et al. [17] investigated matrix cracking deflection at the interphase of the SiC/SiC minicomposite with a pyrocarbon (PyC) interphase. The deflection of matrix cracking depends on the interface bond strength and interphase type. Sauder et al. [18] investigated the tensile and cyclic loading/unloading behavior of the SiC/SiC minicomposite with different interphases. The interphase thickness and the fiber surface roughness affect the tensile nonlinear and fracture strain of the minicomposite due to different interface debonding conditions. Yu et al. [19] investigated the effect of SiC coating thickness on the mechanical behavior of the SiC/SiC composite. The flexural strength of the SiC/SiC composite initially increased with the increase in SiC coating thickness, reached a peak value, and then decreased rapidly; however, the bending modulus increased with the increase in SiC coating thickness. He et al. [20] investigated the tensile behavior of the SiC/SiC minicomposite with different interphase thicknesses and matrix volume fractions. With the increase in the thickness of the interface layer, the tensile strength of the minicomposite increased, and the fiber’s pull-out length also increased; however, at the same interface layer thickness, a higher matrix volume fraction decreased the composite’s tensile strength and toughness. Mei et al. [21] investigated the effect of heat treatment on the strength and toughness of C/SiC composites with different pyrolytic carbon (PyC) interphase thicknesses. Kabel et al. [22] investigated the relationship between the PyC interphase properties and debonding shear strength of the SiC/SiC composite. Zhang et al. [23] established a micromechanical constitutive model to predict the tensile damage and fracture behavior of SiC/SiC minicomposites. In the research mentioned above, the relationship between the interphase type and thickness and their effect on the mechanical behavior of the SiC/SiC composite, especially tensile and shear strength, are examined. However, during the application of CMC components, the design stress was considerably lower than the composite’s tensile strength [24]. It is necessary to establish the relationship among the nonlinear mechanical behavior, microdamage mechanisms, and interphase properties of the C/SiC composite [25].

Minicomposites (unidirectional composites containing a single bundle of fibers) are used to study the nonlinear behavior of fiber-reinforced CMCs for different damage mechanisms [25,26,27]. The objectives of this paper are to evaluate the tensile damage and fracture behavior of C/SiC minicomposites with single- and multiple-layer interphases and to establish the relationship among macrotensile nonlinear behavior, microdamage mechanisms, and interphase properties. The effect of the interphase on tensile damage and fracture mechanical behavior of the C/SiC minicomposite is discussed. To analyze the microdamage mechanisms, in situ damage observations of a notched C/SiC minicomposite are performed using the digital image correlation (DIC) method. The evolution of matrix cracking and propagation is analyzed. Comparisons of fiber pull-out lengths in the single-layer and multi-layer interphase are conducted.

## 2. Materials and Experimental Procedures

**Figure 1:**
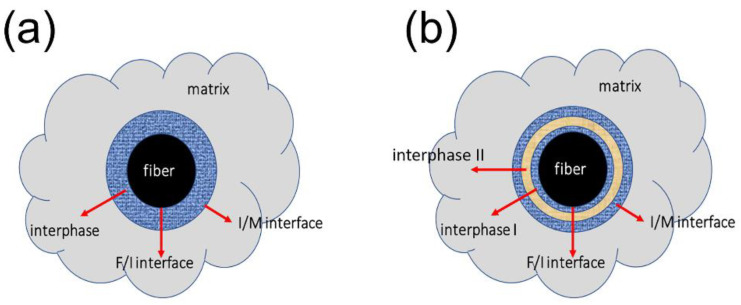
The interface and interphase between the fiber and matrix: (**a**) single-layer interphase; (**b**) multi-layer interphase. (F—fiber, M—matrix, and I—interphase).

T-700^TM^ 12k carbon fiber reinforced silicon carbide minicomposites (C/SiC mini-CMCs) were fabricated using the chemical vapor infiltration (CVI) method. Carbon fiber was wound on the graphite tooling and fixed in a chemical vapor deposition (CVD) furnace. The fiber preform was divided into fiber filaments (FF) and non-woven cloth (NC). Different interfaces were prepared by controlling the composition or sequence of reactive gases flowing into the CVD furnace. The interphase thickness and matrix densification of composites with the same interface were changed by controlling the deposition time and matrix densification time. For each type of fiber preform, the interphase can be divided into a 6 h PyC layer, an 18 h PyC layer, and a 4-layer PyC-SiC, as shown in Table 1. Figure 1 shows the scheme of the single-layer and multi-layer interphase.

**Figure 2:**
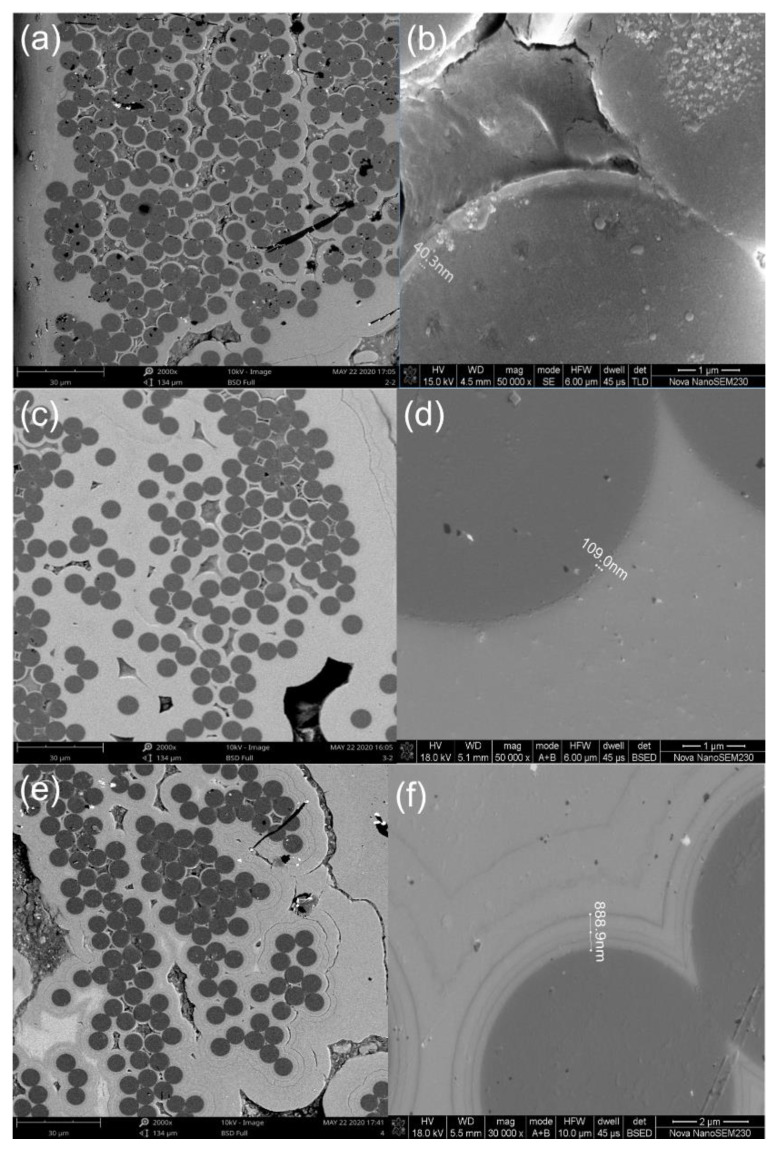
C/SiC minicomposite with different interphases: (**a**) Type I interphase; (**b**) the interphase thickness of Type I interphase; (**c**) Type II interphase; (**d**) the interphase thickness of Type II interphase; (**e**) Type III interphase; and (**f**) the interphase thickness of Type III interphase.

Table 2 shows detailed information regarding the processing method of C/SiC minicomposites with different interphases. Three types of interphases were fabricated for the C/SiC minicomposite, including:

- Type I Interphase, i.e., the 6 h PyC single-layer interphase. Using propylene as a carbon source precursor, argon as dilution, and protective gas, the PyC single-layer interphase was deposited at approximately 1000 °C with a deposition pressure of 200 Pa and a deposition duration of 6 h. The thickness of the PyC single-layer interphase was approximately 40.3 nm, as shown in Figure 2a,b.
- Type II Interphase, i.e., the 18 h PyC single-layer interphase. The PyC deposition temperature was approximately 1000 °C with a duration of 18 h. The thickness of the PyC single-layer interphase was approximately 109 nm, as shown in Figure 2c,d.
- Type III Interphase, i.e., the (PyC-SiC)_4_ interphase. In the CVD process, the mixed gas of propylene–argon and MTS–hydrogen–argon was alternately introduced into the CVD furnace. The deposition temperature of the PyC interface was approximately 1000 °C, and the deposition temperature of the SiC interface was approximately 1050 °C. (PyC/SiC)_n_ multi-layer interfaces were obtained by controlling the deposition time and alternating times. The first layer of the (PyC/SiC)_n_ multi-layer interface is the PyC layer, and the last layer is the SiC layer. The thickness of the (PyC-SiC)_4_ interphase was approximately 888.9 nm, as shown in Figure 2e,f.

Table 3 shows the detailed data regarding the dimensions of each sample. The tension tests were carried out using the Instron Mechanical test with a 0.5 mm/min loading rate and a 2 kN maximum load. For the NC_2 C/SiC minicomposite, a short notch was cut on the specimen using diamond wire cutting.

## 3. Results and Discussions

In this section, the tensile behavior of C/SiC minicomposites with a single PyC layer and multi-layer (PyC/SiC)_4_ interphase is analyzed. The evolution of matrix cracking of the C/SiC minicomposite with a notch is analyzed using the DIC method. The fiber pull-out lengths in the C/SiC minicomposites with single-layer and multi-layer interphases are compared.

### 3.1. Tensile Behavior of C/SiC Minicomposites

**Figure 3:**
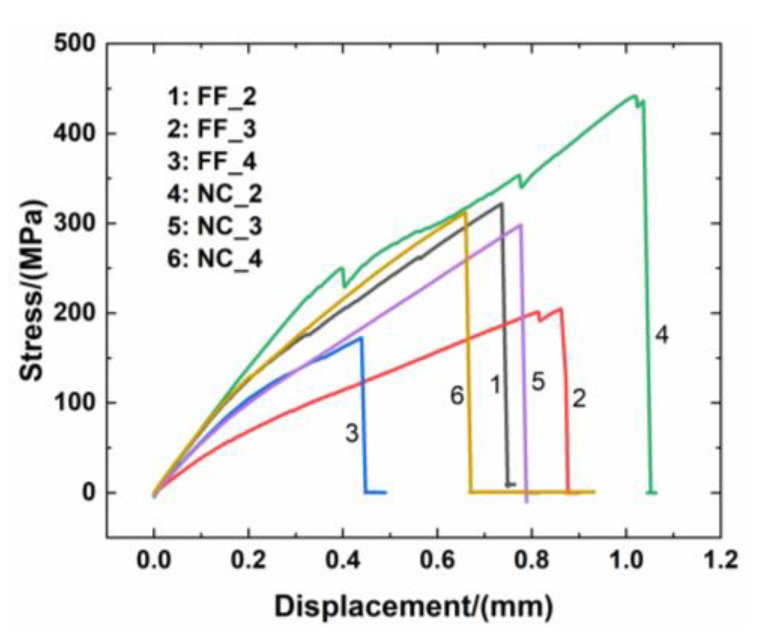
Load–displacement curves of C/SiC minicomposites with different interphase.

Figure 3 shows the stress–displacement curves of C/SiC minicomposite samples with different interphases and reinforcements. From the curves, all C/SiC minicomposites exhibit nonlinear characteristics and brittle fracture behavior. For the C/SiC composite without the interphase, the tensile behavior of the composite exhibits linear fracture behavior as the cracking in the matrix penetrates through the fiber.

- For FF_2 C/SiC with the 6 h single-layer PyC interphase, the tensile curve of the minicomposite exhibited linear elastic behavior until the proportional limit stress (PLS) of approximately *σ*_PLS_ = 95 MPa was reached, and the tensile curve appeared nonlinear due to the matrix cracking and interface debonding until the applied stress of approximately *σ* = 176 MPa was reached. Then, the tensile curve displayed linear elastic behavior again until the tensile fracture at the strength of approximately *σ*_UTS_ = 321.9 MPa occurred.
- For FF_3 C/SiC with the 18 h single-layer PyC interphase, the tensile curve of the minicomposite exhibited linear elastic behaviour until reaching the proportional limit stress (PLS) of approximately *σ*_PLS_ = 40 MPa, and the tensile curve appeared nonlinear due to matrix cracking and interface debonding until reaching the applied stress of approximately *σ* = 120 MPa. Then, the tensile curve displayed linear elastic behavior again until the tensile fracture at the strength of approximately *σ*_UTS_ = 204.6 MPa occurred. Before the tensile fracture occurred, the tensile curve showed an obvious zig-zag pattern due to the fibers’ fracture.
- For FF_4 C/SiC with the 4-layer PyC-SiC interphase, the tensile curve of the minicomposite exhibited linear elastic behavior until the proportional limit stress (PLS) of approximately *σ*_PLS_ = 89 MPa was reached, and the tensile curve appeared nonlinear due to matrix cracking and interface debonding until reaching the applied stress of approximately *σ* = 138 MPa. Then, the tensile curve displayed linear elastic behavior again until the tensile fracture at the strength of approximately *σ*_UTS_ = 172.2 MPa occured. Under tensile loading, the tensile curve did not show zig-zag behavior.
- For NC_2 C/SiC with the 6 h single-layer PyC interphase, the tensile curve of the minicomposite exhibited linear elastic behavior until the proportional limit stress (PLS) of approximately *σ*_PLS_ = 196 MPa was reached, and with an increasing load, the zig-zag behavior occured at the applied stresses of *σ* = 249, 353, and 441 MPa, due to matrix cracking and fiber fracture. The composite tensile fracture occured at the strength of approximately *σ*_UTS_ = 441.5 MPa.
- For NC_3 C/SiC with the 18 h single-layer PyC interphase, the tensile curve of the minicomposite exhibited linear elastic behavior until the proportional limit stress (PLS) of approximately *σ*_PLS_ = 72 MPa was reached, and the tensile curve appeared nonlinear due to matrix cracking and interface debonding until reaching the applied stress of approximately *σ* = 141 MPa. Then the tensile curve displayed linear elastic behavior again until the tensile fracture at the strength of approximately *σ*_UTS_ = 298.4 MPa occurred. There was no zig-zag pattern under tensile loading.
- For NC_4 C/SiC with the 4-layer PyC-SiC interphase, the tensile curve of the minicomposite exhibited linear elastic behavior until the proportional limit stress (PLS) of approximately *σ*_PLS_ = 94 MPa was reached, and with increasing load, zig-zag behavior occured at the stress of *σ* = 130 MPa, mainly due to matrix cracking and a continually increasing load. The tensile curve showed nonlinear behavior until the tensile fracture at the strength of approximately *σ*_UTS_ = 311.9 MPa occurred.

Table 4 displays the experimental results of the tensile data. It can be observed that there is no evident influence on the strength utilization of carbon filaments in the process of weaving the cloth from the original carbon fiber. The single-layer PyC interphase exhibits much better mechanical properties than the multi-layer interface does if the reasonable thickness of the PyC layer is effectively controlled. The function of the interface is mainly exerted through the thickness of the interphase (either the single-layer PyC interphase or the multi-layer (PyC-SiC) interphase in this case) on the mechanical property. The composite’s tensile strength was the highest for the fiber filament and now-woven cloth reinforced C/SiC minicomposites with a single-layer PyC interphase, i.e., *σ*_UTS_ = 321.9 MPa for FF_2 and *σ*_UTS_ = 441.5 MPa for NC_2 C/SiC minicomposites. The thickness of the single PyC interphase was approximately 40.3 nm. However, when increasing the PyC interphase thickness from 40.3 to 109 nm, the composite’s tensile strength decreased to *σ*_UTS_ = 204.6 MPa for the fiber filament reinforced C/SiC minicomposite and to *σ*_UTS_ = 298.4 MPa for the non-woven cloth reinforced C/SiC minicomposite. The degradation of the composite’s strength is due to the damage to the fiber’s strength under a longer deposition time for interphase thickness at an elevated temperature. For the multiple layer (PyC-SiC) interphase, the composite’s tensile strength for the non-woven reinforced C/SiC minicomposite was much higher than that of the fiber filament reinforced C/SiC minicomposite, i.e., *σ*_UTS_ = 311.9 MPa versus *σ*_UTS_ = 172.2 MPa, which indicates that the multiple-layer interface cannot deflect the matrix cracking for the unidirectional C/SiC composite with a short fiber pull-out length.

Due to the brittleness of the matrix, the fracture of the C/SiC minicomposites causes progressive damage, accumulating from the microscale to the macroscale, which can be generally divided into several steps, namely, evolution of matrix cracking, fiber/matrix interface debonding, fiber fracture, and fiber pull-out [28,29,30,31,32,33,34]. Due to the mismatch of the thermal expansion coefficient between the carbon fiber and SiC matrix, microcracks in the matrix first appear during the fabrication process [35,36]. With increasing load, matrix cracks propagate forward to the fiber/matrix interfaces and then to the fibers. The density of matrix cracking increases with applied stress [37]. Li [38] developed a micromechanical approach to predict the matrix multiple-cracking evolutions of mini- and uni-directional and 2D plain-woven SiC/SiC composites and established the theoretical relationship between matrix cracking and the composite’s components. It can be suggested that the composite exhibits extended nonlinear stress–strain mechanical behavior related to progressive damage of matrix microcracking, the bundle/matrix interface, and inter-bundle debonding, as well as thermal residual and mechanical stress relaxation. With respect to the damage mechanism, three or four stages may occur under tensile load:

- Stage I, an elastic response coupled with partial re-opening of thermal microcracking.
- Stage II, multiple matrix microcracking perpendicular to the applied loading.
- Stage III, crack opening and related fiber/matrix and mostly bundle/matrix interfaces and inter-bundle debonding.
- Stage IV, progressive transfer of load to the fiber and gradual fiber failure until composite failure/fracture.

**Figure 4:**
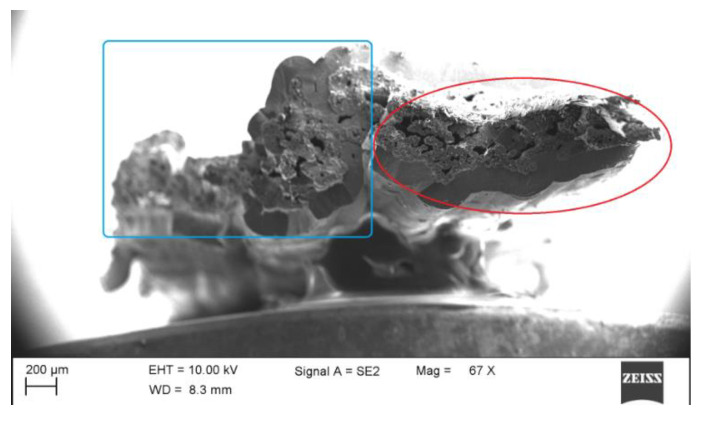
The fracture section of sample NC_2.

**Figure 5:**
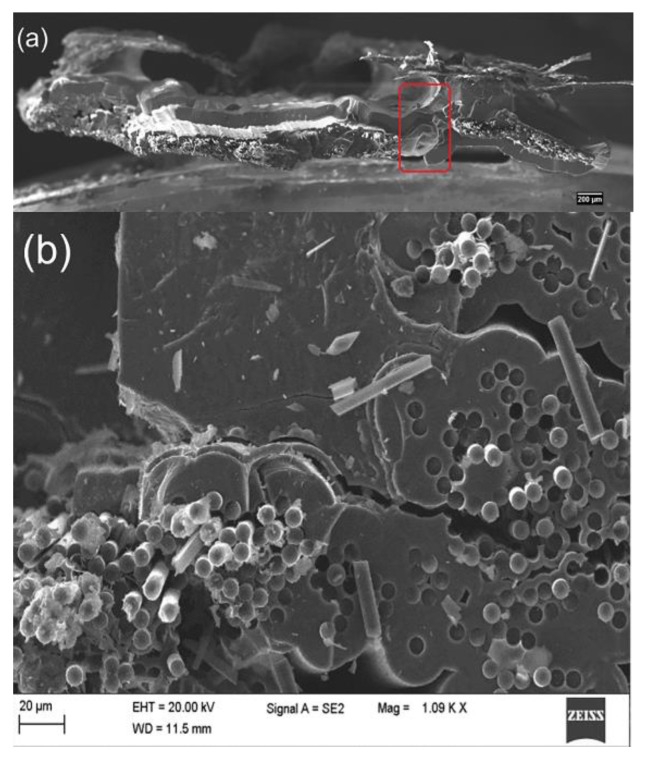
Morphologies and topographies of the fracture section of sample FF_2: (**a**) overall morphology of fracture surface; (**b**) local morphology of amplification.

In addition, it is worth noting that for sample NC_2, the load–displacement curve exhibited zig-zag behavior, which means that part of the carbon fiber filaments were broken first, and the remaining the filaments continued to bear the increasing load. In fact, this sample was made up of two carbon fiber bundles, which became stuck together during the process of CVI SiC densification, as shown in Figure 4. Two bundles of carbon fibers are marked by a blue rectangle box and a red ellipse box. From the fracture section, a fracture step between the bundles can be clearly seen, which means that the two bundles did not break synchronously under the load. This phenomenon can also be found in sample FF_2, which is just one bundle of carbon fiber as reinforcement. From the curve of FF_2, some discontinuous changes can be observed, which indicates that all of the filaments in the whole bundle could not simultaneously carry the load. It is worth mentioning that bundle integrity plays an important role in the mechanical properties. Lacking bundle integrity, sample FF_2 could not take the load as a whole, and the fracture of the minicomposite exhibited progressive damage during the tensile test, which implies that partial fiber filaments fracture first, and then the remaining filaments are disrupted under further loading, as shown in Figure 5.

### 3.2. Tensile Behavior of Notched C/SiC Minicomposites

**Figure 6:**
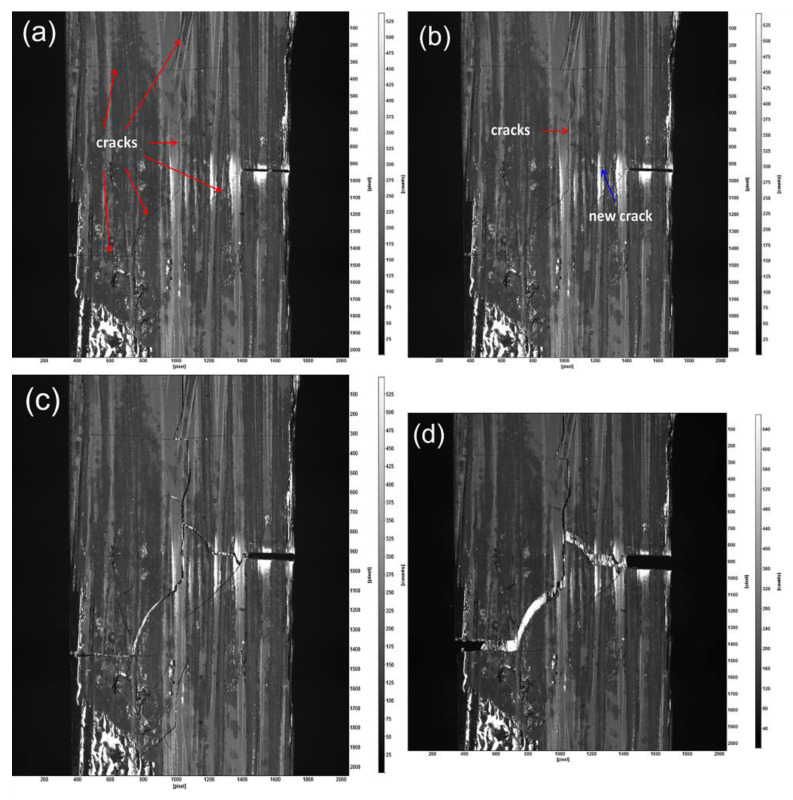
The whole fracture process of sample NC_2: (**a**) microcracks in the matrix; (**b**) a new crack initiated along the notch; (**c**) cracks opening and coalescence under tension; (**d**) complete fracture of the whole bundle.

Digital image correlation (DIC) is a non-contact optical technique that is capable of measuring displacement and calculating strain fields, and it has been proven to be a reliable method for the study of material deformation and crack propagation. Figure 6 shows the fracture process of sample NC_2 with a single PyC interphase according to the sequence of fracture progression.

Via monitoring with a high-resolution camera, a series fracture processes were recorded and obtained. Under uniaxial tensile loading, the DIC system collects a set of scenes involving matrix opening, fiber fracture, and bundle fracture. The major process can be described as follows:

- At the beginning of the test, there are a large amount of initial microcracks existing in the matrix, which result from the CTE mismatch between the carbon fiber and the SiC matrix, especially for the thick matrix. These cracks differ in length, shape, and orientation, being aligned parallel to the fiber (longitudinal) or perpendicular to the fiber (transverse). One of the microcracks even runs through the width of the sample, as shown in Figure 6a.
- With increasing load, a new microcrack begins to nucleate near the tip of the notch. This crack grows along the direction of the notch, accompanied by the matrix open and fiber breakage. In this field of view, there occurs progressive growth and coalescence of cracks, within which, the two cracks (indicated as a red arrow and a blue arrow) seem to become the main cracks, and others display a somewhat obvious change under tension, as shown in Figure 6b. The stress near the tip of cracks relaxes along with this zone, which also means that this zone is the fracture plane, as shown in Figure 6c.
- When all fibers break, the sample fractures along the main crack growth path, as shown in Figure 6d.

From the observation of the failure mechanism, it appears that the stress in the tip of the crack was preferentially induced and caused matrix cracking, interface debonding, and fibers fracture.

**Figure 7:**
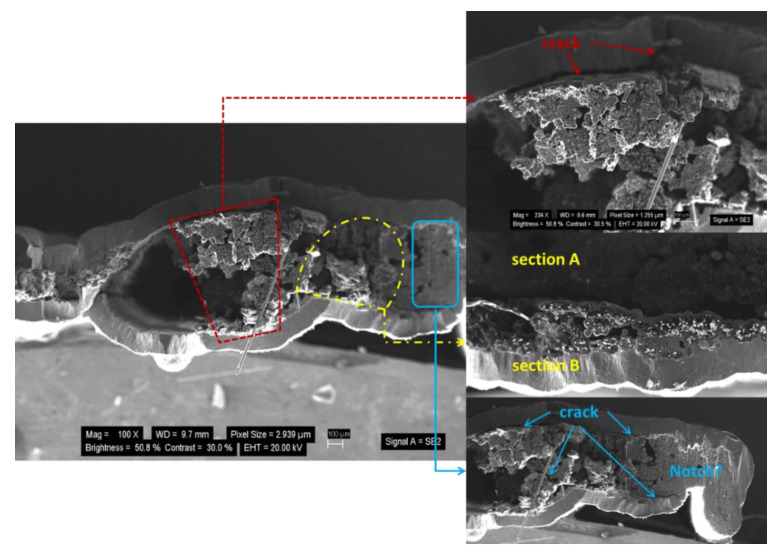
Fracture morphologies of sample NC_2.

At the microscale, the morphologies of the fracture section distinctly display the fracture process of sample NC_2 and provide clues regarding the major factors causing the damage, allowing us to identify the relevant mechanism of the fractures, as shown in Figure 7. It is clear that the fracture caused weak regions around the notch, such as the initial microcracks in the matrix. In addition, it is likely that the rich matrix region (indicated by the red dotted line) among the bundles developed into long cracks, which may have induced stress relaxation. There existed several macrocracks between the thick matrix and fiber filaments rather than a considerable number microcracks, which indicates that the thick matrix is detrimental to the strength of materials. Furthermore, debonding between the matrix and fiber bundle gave rise to degradation of strength utilization, as the matrix does not share the load. It is worth mentioning that the SiC matrix was fabricated three times by the CVD process, in which the matrix after the first two CVDs was much thinner than that in the third matrix. The main reason for this is the fact that the bonding between the fiber and SiC matrix after the first two CVDs was significantly higher than that between the matrix in the third CVD and the first two CVDs. Due to the densification segmentation of the CVD process, the microstructure of the SiC matrix was discontinuous, which means that it was prone to become the potential debonding plane. The whole fiber bundle was divided into lots of relatively independent units, which led to the poor unity of the fiber bundle. The sample exhibited brittle failure, and its fracture section was smooth and flat. The fracture process involved the interaction of multiple factors, such as excessively applied stress in the matrix, weak debonding, and pulling out derived from the PyC interphase, as well as poor integrity of the fiber bundle.

### 3.3. Fiber Pull-out of C/SiC Minicomposite

**Figure 8:**
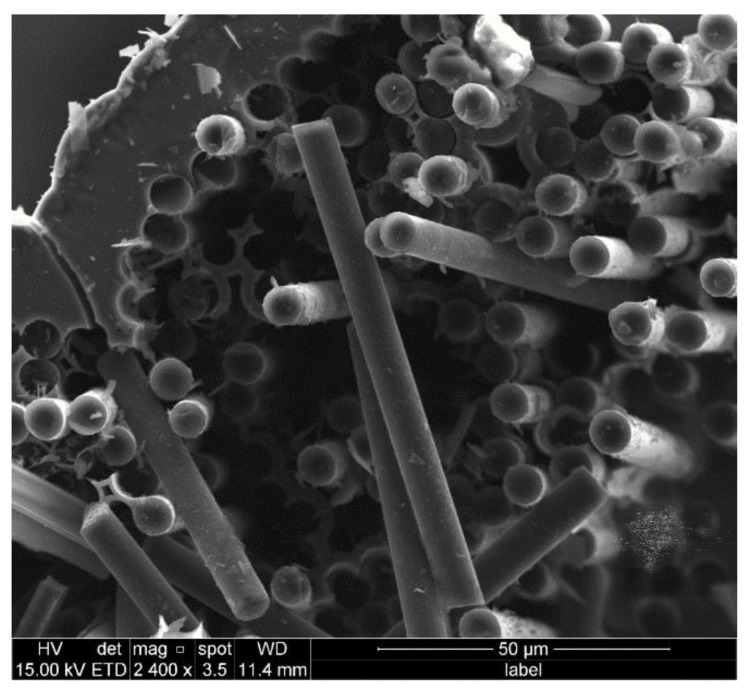
Fiber fracture morphology of sample FF_2.

**Figure 9:**
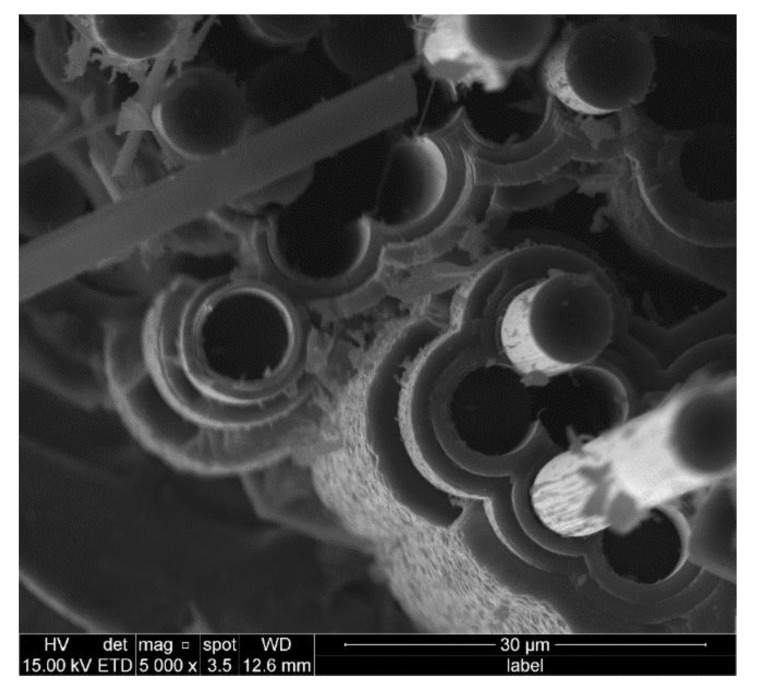
Fiber fracture morphology of sample FF_4.

From Figure 8 and Figure 9, a large number of fibers pulling out of the samples with both single-layer and multi-layer interphase can be clearly found. Furthermore, some clearages of fibers break-off and cavities after fiber pull-out remain on the fracture section, which indicates the failure with extensive fiber pull-out occurs. In addition, the smooth surface of the fibers can be observed from the morphologies, which are highly similar to that of the original T-700^TM^ 12k carbon fiber as reinforcement. All the above observations prove that the cracks initiate at the interface between the fiber and the interphase for the sake of the weak bonding. In other words, the bonding strength between fiber and interphase is much less than that between interphase and matrix, even less than that among the sublayers in the multi-layer interphase. According to the above discussion, these weak zones will have the priority to release the principal stress in the samples, which leads to the fiber debonding and sliding under the uniaxial tension load.

## 4. Conclusions

In this paper, two different reinforcement types (i.e., fiber filament and non-woven cloth) of C/SiC minicomposites with a single-layer PyC interphase and a multiple-layer (PyC/SiC) interphase were examined. The composites exhibited different mechanical behavior under tensile loading for different proportional limit stresses and tensile strengths. The notched C/SiC minicomposite was observed using in situ DIC monitoring under tensile loading to analyze matrix multiple cracking propagations. A comparison of fiber pull-out lengths in single-layer and multi-layer interphases was conducted.

- There is no evident influence on the strength utilization of carbon filaments in the process of weaving cloth from the original carbon fiber. The single-layer PyC interphase exhibits much better mechanical properties than the multi-layer interphase does if the reasonable thickness of the PyC layer is effectively controlled.
- The bundle integrity plays an important role in the mechanical properties as well as in the proper interphase. Lacking bundle integrity, the C/SiC minicomposite cannot take the load as a whole, and the fracture of the minicomposite exhibits multi-stage damage during the tension test, which implies that some of the fiber filaments result in failure, and remaining filaments are disrupted under further loading.
- A large number of fibers pulling out of the samples with both single-layer and multi-layer interphases can be clearly observed. Some clearages of fiber break-off and cavities after fiber pull-out remain on the fracture section, which indicates the failure when extensive fiber pull-out occurs.

## Figures and Tables

**Table 1:** Overall information regarding all of the samples.

Material		1st Group	2nd Group
Carbon fiber type		T-700^TM^-12k	
Reinforcement		fiber filament (FF)	non-woven cloth (NC)
Interphase	2#	6 h PyC single layer	
	3#	18 h PyC single layer	
	4#	4-layer PyC-SiC multi-layer	
Matrix		CVI-derived SiC	

**Table 2:** The detailed processing parameters of the samples in each group.

Sample Number	Interface Type	Interface Processing	SiC Matrix Deposition
2#	6 h PyC single layer	Temperature: 1000 °C Pressure: 200 Pa Duration: 6 h Gas precursor: propylene Gas flow rate: 160 mL/min Dilute gas and flow rate: argon with 400 mL/min	Stage1: 1050 °C for 50 h Stage2: 1100 °C for 100 h
3#	18 h PyC single layer	Temperature: 1000 °C Pressure: 200 Pa Duration: 18 h Gas precursor: propylene Gas flow rate: 160 mL/min Dilute gas and flow rate: argon with 400 mL/min	Stage1: 1050 °C for 50 h Stage2: 1050 °C for 100 h Stage3: 1100 °C for 100 h
4#	4-layer PyC-SiC multi-layer	Interphase: (Py-SiC)_4_ For PyC, Temperature: 1000 °C Duration: 3 h Gas precursor: propylene Gas and flow rate: 160 mL/min C3H6 and 400 mL/min Ar For SiC, Temperature: 1050 °C Duration: 3 h Gas precursor: Methyltrichlorosilane Gas and flow rate: 160 mL/min H_2_ as carrier gas and 200 mL/min Ar as dilute gas	Stage1: 1050 °C for 100 h Stage2: 1100 °C for 100 h

**Table 3:** The sample dimensions for different C/SiC minicomposites.

Sample Number	Total Length/mm	Width/mm	Gauge Length/mm
FF-2	67	7	10
FF-3	67	7.5	10
FF-4	67	7	10
NC-2	76	5.5	20
NC-3	83	4	20
NC-3	86	4	20

**Table 4:** The tension strength of each sample.

Samples	Max Load, N	Cross-Section/Net-Section Area, mm^2^	Strength, MPa
FF_2	727.537	2.26	321.9
FF_3	840.91	4.11	204.6
FF_4	518.41	3.01	172.2
NC_2	1028.73	2.33	441.5
NC_3	713.08	2.39	298.4
NC_4	695.58	2.23	311.9

## Data Availability

The data used to support the findings of this study are available from the paper.
